# Prolonged secretion of cortisol as a possible mechanism underlying stress and depressive behaviour

**DOI:** 10.1038/srep30187

**Published:** 2016-07-22

**Authors:** Dong-dong Qin, Joshua Rizak, Xiao-li Feng, Shang-chuan Yang, Long-bao Lü, Lei Pan, Yong Yin, Xin-tian Hu

**Affiliations:** 1 Key Laboratory of Animal Models and Human Disease Mechanisms of the Chinese Academy of Sciences & Yunnan Province, Kunming Institute of Zoology, Chinese Academy of Sciences, Kunming, Yunnan, 650223, China; 2 Kunming Primate Research Center, Chinese Academy of Sciences, Kunming Institute of Zoology, Chinese Academy of Sciences, Kunming, Yunnan, 650223, China; 3 Department of Rehabilitation Medicine, the Fourth Affiliated Hospital of Kunming Medical University, Kunming, Yunnan, 650021, China; 4 CAS Center for Excellence in Brain Science, Chinese Academy of Sciences, 320 Yue Yang Road, Shanghai, 200031, China

## Abstract

Stress is associated with the onset of depressive episodes, and cortisol hypersecretion is considered a biological risk factor of depression. However, the possible mechanisms underlying stress, cortisol and depressive behaviours are inconsistent in the literature. This study examined the interrelationships among stress, cortisol and observed depressive behaviours in female rhesus macaques for the first time and explored the possible mechanism underlying stress and depressive behaviour. Female monkeys were video-recorded, and the frequencies of life events and the duration of huddling were analysed to measure stress and depressive behaviour. Hair samples were used to measure chronic cortisol levels, and the interactions between stress and cortisol in the development of depressive behaviour were further evaluated. Significant correlations were found between stress and depressive behaviour measures and between cortisol levels and depressive behaviour. Stress was positively correlated with cortisol levels, and these two factors interacted with each other to predict the monkeys’ depressive behaviours. This finding extends the current understanding of stress/cortisol interactions in depression, especially pertaining to females.

Depression is a common mental disorder characterized by an unremitting despondent mood, which manifests with symptoms of anhedonia, feelings of worthlessness, helplessness and hopelessness, decreased appetite, difficulty concentrating and an overall low “spirit”^1^. These symptoms may lead to long-standing or chronic problems and eventually to an inability to function in everyday life. In addition, depressed patients have a greater risk of developing coronary artery disease and type 2 diabetes^2^. The World Health Organization (WHO) currently lists depression as the fourth major cause of disability and estimates that depression will become the second leading cause of disability after heart disease by the year 2020^3^.

Epidemiological studies have indicated that depression occurs nearly twice as frequently in women than in men, and this prevalence has been ascribed to several biological processes, which include genetically determined vulnerabilities, hormone changes associated with reproductive function, and hypersensitivity to hormones that precipitate depression^4^. Hormonal fluctuations related to reproductive changes, such as hormonal changes during puberty or postpartum, have been found to increase the risk of onset of major depression in women from early adolescence until their mid-50s^5^. However, other experiences related to changes in sex hormones (pregnancy, menopause, use of oral contraceptives, or hormone replacement therapy) in women have not been found to significantly influence depressive symptoms. This suggests that, in women, other complex factors contribute to the onset of depression and that the key to understanding the higher rates of depression in women compared to men may depend on an investigation the combined effects of biological vulnerabilities and environmental determinants^6^.

Stress has become a major focus of psychiatric epidemiology regarding depression^7^ since Holmes and Rahe’s pioneering work correlating stressful life events and illness^8^. It has been reported that depressed patients generally experience higher levels of stressful life events prior to the onset of depressive episodes compared to controls^9^. For example, undesirable life events and friendship difficulties exert independent and additive effects on the probability of developing depression in children aged 7–16 years^10^. In addition, studies of adult subjects also reported that depressed patients have higher rates of stressful events than age- and sex-matched controls^11^. In fact, stressful life events not only influence the first onset of depression but also affect the severity, remission or relapse of depressive episodes^7^. Stress has also been related to weakened antidepressant responses in depressed patients^12^.

A major focus in investigations of the relationship between stress and depression is the role of the hypothalamic-pituitary-adrenal (HPA) axis, both as a marker of stress responses and as a mediator of additional downstream pathological consequences. Dysfunction of the HPA axis has been considered a state hallmark of human depression^13^, which has also been reported in depressed monkeys^14^,^15^. During stress, the HPA axis is involved in regulating the secretion of corticotropin-releasing hormone-mediated glucocorticoids. The secretion of glucocorticoids has both adaptive and adverse effects. The acute release of glucocorticoids is thought to enhance cardiovascular function, mobilize carbohydrates and inhibit organism growth, reproduction and immunological responses^13^. However, the adaptive advantages of glucocorticoid secretion are limited to its acute rather than chronic release. Chronic elevation of glucocorticoids is considered detrimental to health. For example, research in rats and nonhuman primates has found that exposure to excessive glucocorticoids damages the hippocampus^16^, which leads to negative outcomes such as the regression of dendritic processes, an inhibition of neurogenesis, an inability to survive insults such as a stroke or seizure, and the promotion of neurotoxicity.

Cortisol hypersecretion has also been considered a biological risk factor of depression^17^. As early as 1962, it was reported that patients diagnosed with depression hyper-secreted cortisol^18^. It is widely hypothesized that chronic stress leads to an increased secretion of cortisol and results in depression. In fact, it has been shown that cortisol hypersecretion observed in depressed patients was directly related to the stress that the subjects experienced^19^. However, there is also evidence that has shown that, despite cortisol’s sensitivity to stress, cortisol does not mediate vulnerability to depression^20^. Some of the factors that are difficult to measure, thus leading to these inconsistencies, include variability in human lifestyles^21^ and measures of stressful events and depressive symptoms^22^ that are difficult to control in a complex society. These inconsistencies are compounded by the various cortisol measurement techniques used in many previous studies^18^,^23^,^24^. Therefore, it is not surprising that the interrelationships and interactions among these two important variables in depression remain undefined.

To further understand the physiological mechanism between stress and depression, better controlled animal studies in the laboratory are required. Nonhuman primates, especially rhesus macaques, provide an excellent model because they share a number of similarities with humans. First, the brain structure of rhesus macaques is similar to that of humans^25^, which suggests comparable neural processing of stress. Second, they secret cortisol in response to stress, similar to humans^26^. Third, macaques have a strict social structure, a dependence on social relationships, an ability to engage in complex cognitive processes, a range of affective expressions, and, most importantly, they exhibit a similar depressive response to that of humans^15^.

Furthermore, the living environments of macaques, unlike that of humans, can be tightly controlled, which can provide an easier platform to quantify data and objectively evaluate the physiological mechanisms underlying stress and depression^27^. The present study investigated the behaviours of female rhesus macaques in a controlled setting to clarify the physiological mechanism underlying stress and depressive behaviour because females may have a higher vulnerability to depression.

## Materials and Methods

### Subjects

To explore the possible mechanisms underlying stress and female depression, the monkeys included in this study met the following three criteria. First, they were females and were reproductively mature, as women begin to exhibit higher vulnerability to depression than men after sexual maturity. Second, they had lived in their respective social groups for at least one year prior to the initial observation to ensure the stability of social hierarchy. Third, they were not pregnant and had not given birth in the last year, to avoid the effects of pre-partum or post-partum factors. Ultimately, only 41 females met these requirements among the 2,400 monkeys living in the Kunming Primate Research Center of the Chinese Academy of Sciences and were selected for this study.

The monkeys varied in age from 6 to 21 years (10.290 ± 3.551 years) and were housed in 15 different breeding colonies. Each colony contained 2–4 female monkeys and 1 alpha male, which lived in a connected indoor (261 × 246 × 258 cm)-outdoor (267 × 266 × 267 cm) cage. All animals were given commercial monkey biscuits twice per day and were fed with fruits and vegetables once daily. Tap water was available *ad libitum*.

The care and treatment of the monkeys was in strict accordance with the guidelines for the National Care and Use of Animals approved by the National Animal Research Authority (P.R. China) and the Institutional Animal Care and Use Committee (IACUC) of the Kunming Institute of Zoology (approval ID SYDW20111230001-1). All possible efforts were made to minimize the monkeys’ suffering. For example, hair samples were taken from the back of the monkey’s neck using an electric razor (no anaesthetics were used). Routine veterinary care was provided by professional keepers and veterinarians. No animals were sacrificed as a result of this study.

### Observational Platform

Animal behaviours were video recorded using a focal follow technique^28^ and were then analysed to calculate the frequencies of life events that monkeys experienced and to determine the duration of exhibited depressive behaviours. After completing the video recordings, hair samples were obtained to measure cortisol levels. Next, the interrelationships among life events, cortisol and depressive behaviours were analysed, and the interactions between life events and cortisol in the development of depressive behaviour were further assessed.

### Behaviour sampling

The behavioural data for all monkeys were collected at the same time of the year, which was not during the monkeys’ breeding period. Before video recording, the monkeys had seven days to become familiar with observers and cameras, and during recording, the observers kept as far away as possible (at least five metres) from the monkeys’ cage to avoid disturbing them. According to the DSM-V diagnostic criteria for depression, the behaviours should be collected over a period of no less than two weeks. Therefore, we collected 14 1-hour recordings on 14 consecutive days for each monkey; half were collected in the morning (9:00–11:00), and the other half were collected in the afternoon (14:00–16:00).

### Depressive behaviour

Huddling was used as the behavioural indicator of depression and this has been considered the core posture that reflects depressed moods in monkeys^15^. It is defined as a foetal-like, self-enclosed posture, with the head at or below the shoulders during the awake state (i.e., when the monkey’s eyes are opened), accompanied by a relative lack in responsiveness to environmental stimuli that other monkeys attend to^15^. The total time spent in the huddle posture was recorded as seconds per hour for each monkey while in body contact with the other monkeys, within arm’s reach, or while sitting alone and were used as one cumulative measure of the monkey’s depressive behaviour.

### Stress measures

The frequencies of conflict behaviours were recorded, including both aggressive and submissive behaviours^14^. Aggressive behaviours included bite, slap, grab, stare threat, open-mouth threat, chase, and forced displacement. Submissive behaviours included scream, scream threat, crouching, fleeing, lip smack, grimace, submissive present, and moving away.

Conflict events that each monkey experienced were divided into four categories: receipt of aggression, displays of submission, displays of aggression, and receipt of submission. The stress that monkeys experienced has been directly related to objective quantities of external stressors (i.e., receipt of aggression) and is critically mediated by internal coping processes (i.e., displays of submission)^29^. Therefore, the receipt of aggression and submissive displays were considered the best behavioural representations of stress, and they were pooled additively into the stressful events category. The other two behaviours (displays of aggression and receipt of submission) were considered unlikely to cause stress, as they have been considered to represent stress-attenuating coping responses rather than a source of stress^29^. These two behaviours were pooled additively and used as non-stress measures.

### Hair sampling and cortisol extraction

Hair samples from all monkeys were collected between 13:30 and 15:00. Before the experiment commenced, the hair on the back of each animal’s neck was shaved using an electric razor without the use of an anaesthetic, with particular attention paid by the technicians not to break or damage the skin. After collecting all of the monkeys’ behavioural data (approximately six months), newly grown hair was sampled, as described above, and the obtained hair samples were placed into small pouches of aluminium foil for protection and stored, as previously described^30^.

Cortisol extraction was performed as previously described^29^. Briefly, hair samples were washed twice in 10 mL of isopropanol (3 minutes each) to remove surface contaminants, dried at 35 °C for 8 hours, and then pulverized using a Retsch ball mill (Retsch M400, Germany) at 26 Hz for 2.5 minutes. Powdered hair (400 mg) was weighed and incubated in 8 mL of methanol at room temperature for 24 hours under slow rotation (230 rpm) to extract the cortisol. The samples were then centrifuged at 8,000 × g for 5 minutes, and 4 mL of the supernatant was pipetted into a centrifuge tube and dried under a stream of nitrogen gas. The precipitated extract was reconstituted with 0.5 mL of phosphate-buffered solution (pH: 7.32) and stored at –20 °C until assayed. The cortisol concentration was quantified by radioimmunoassays (RIAs) at the Department of Nuclear Medicine of the Second Affiliated Hospital of Kunming Medical University using a commercially available kit (Cortisol RIA DSL-2000, USA). The cortisol extraction and RIA analyses were performed in a double-blind manner. Each hair sample was tested three times, and the mean of the three cortisol values was used to reduce measurement error.

### Data analysis

The data analysis was conducted using the SPSS software package (SPSS Inc., Chicago, IL, USA). The normality of the data was determined using the Kolmogorov-Smirnov test. In cases where the data were not normally distributed, standard transformation procedures were used to achieve normality (log10 for life events and depressive behaviour). A Pearson correlation analysis was used to evaluate the relationships of age to life events, hair cortisol levels and depressive behaviour, and this analysis was also used to assess the interrelationships among life events, cortisol and depressive behaviour. The interactive effects between life events and cortisol on depressive behaviour were analysed via a multivariate linear regression analyses, and the differences between control monkeys and depressed monkeys were analysed using a one-way ANOVA. In all analyses, *P*-values were determined from two-tailed tests, with the significance level set at *P* < 0.05. All data are presented as the mean ± SEM (standard error of the mean).

## Results

### Relationships of age to life events, cortisol levels and depressive behaviour

**Figure 1:**
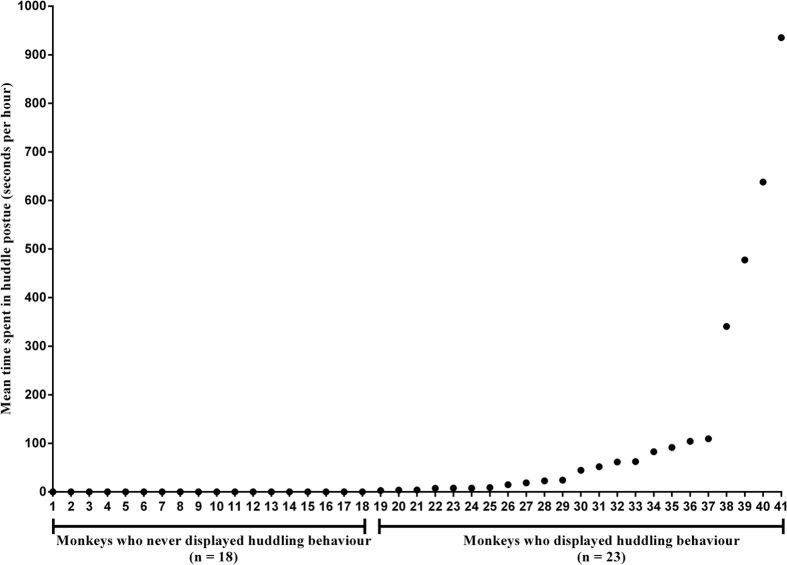
Huddling behaviours. On the x-axis, monkey individuals are divided into two groups: monkeys who never displayed huddling behaviours (n = 18) and monkeys who displayed huddling behaviours (n = 23). The y-axis displays the mean time spent in a huddled posture (seconds per hour).

**Figure 2:**
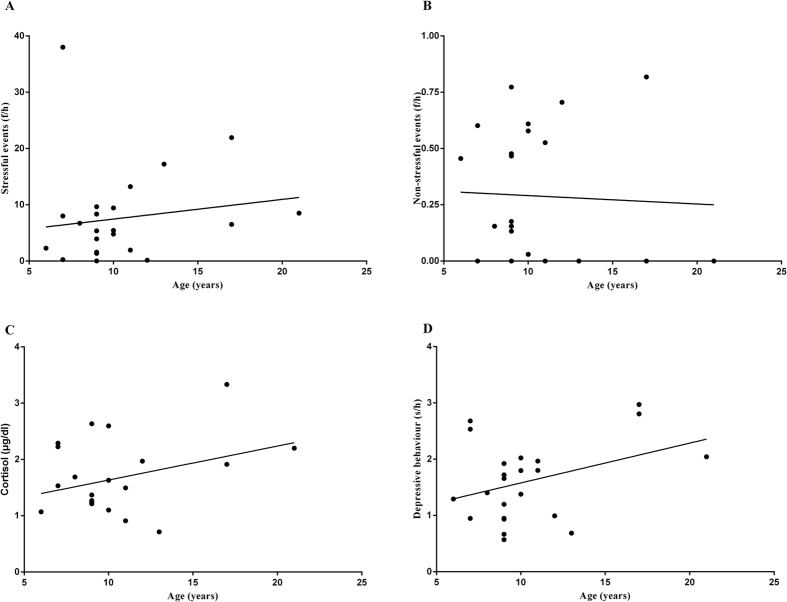
Relationships of age to life events, cortisol levels and depressive behaviour. The x-axis represents the monkeys’ ages (years). The y-axis represents (**A**) the mean frequencies of stressful events that monkeys experienced per hour (f/h); (**B**) the logarithm of the mean frequencies of non-stressful events that monkeys experienced per hour (f/h); (**C**) the mean hair cortisol levels (μg/dl); and (**D**) the logarithm of the mean huddling time that monkeys displayed per hour (s/h).

There were 18 monkeys who never displayed depressive behaviour, and the following correlation analyses were performed for the remaining 23 monkeys (Fig. 1). No significant correlations were found between age and the frequencies of life events experienced by these 23 females. The Pearson correlation coefficients between age and frequencies of animals’ experienced life events are as follows: stressful events (Fig. 2A, *r* = 0.145, *P* = 0.509) and non-stressful events (Fig. 2B, *r* = −0.045, *P* = 0.837). No significant correlations were evident between age and cortisol (Fig. 2C, *r* = 0.338, *P* = 0.115) or between age and depressive behaviour (Fig. 2D, *r* = 0.363, *P* = 0.089).

### Interrelationships between life events, cortisol and depressive behaviour

**Figure 3:**
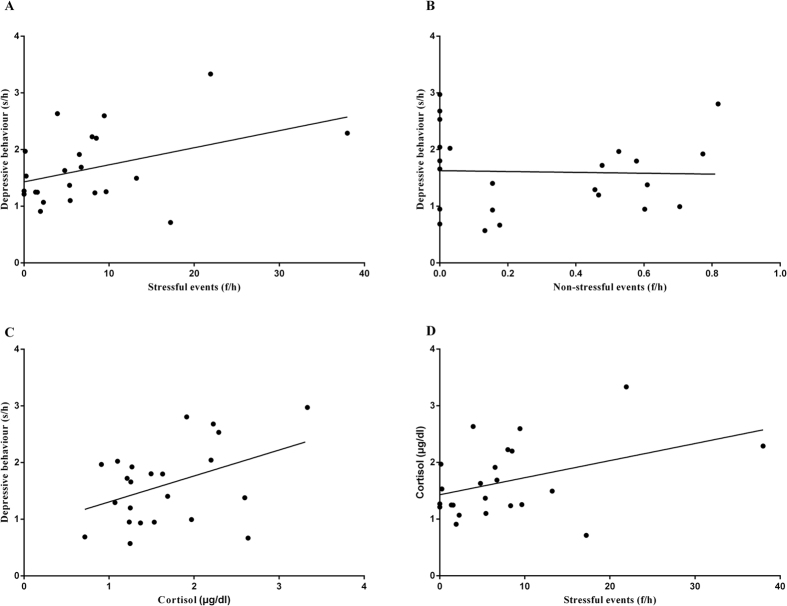
Interrelationships among life events, cortisol and depressive behaviours. (**A**) The x-axis represents the mean frequencies of stressful events that monkeys experienced per hour (f/h). The y-axis represents the logarithm of the mean huddling time that the monkeys displayed per hour (s/h). (**B**) The x-axis represents the logarithm of the mean frequencies of non-stressful events that the monkeys experienced per hour (f/h). The y-axis represents the logarithm of mean huddling time that monkeys displayed per hour (s/h). (**C**) The x-axis represents mean hair cortisol levels (μg/dl). The y-axis represents the logarithm of the mean huddling time that the monkeys displayed per hour (s/h). (**D**) The x-axis represents the mean frequencies of stressful events that the monkeys experienced per hour (f/h). The y-axis represents the mean hair cortisol levels (μg/dl).

Pearson correlations were used to evaluate the interrelationships between huddle behaviour and (i) life events and (ii) cortisol. Stressful events were positively correlated with time spent in the huddle posture (Fig. 3A, *r* = 0.425, *P* = 0.043), whereas no significant relationship to huddle behaviour was found for non-stressful events (Fig. 3B, *r* = −0.032, *P* = 0.885). The same analysis also demonstrated that high levels of cortisol correlated to a longer time spent in the huddle posture (Fig. 3C, *r* = 0.419, *P* = 0.047). Although the correlation between stressful events and cortisol was not very significant, it approached marginal levels of significance (Fig. 3D, *r* = 0.407, *P* = 0.054). However, there was no significant relationship between non-stressful events and cortisol levels (*r* = −0.056, *P* = 0.666).

### Interactive effects between stressful events and cortisol on depressive behaviour

The results of correlation analyses showed that the depressive behaviour was significantly related to both stressful events (Fig. 3A, *r* = 0.425, *P* = 0.043) and cortisol (Fig. 3C, *r* = 0.419, *P* = 0.047). However, the depressive behaviour was not significantly correlated with either age (Fig. 2D, *r* = 0.363, *P* = 0.089) or non-stressful events (Fig. 3B, *r* = −0.032, *P* = 0.885). Cortisol was further found to be marginally related to the stressful events (Fig. 3D, *r* = 0.407, *P* = 0.054) that the monkeys experienced rather than to age (Fig. 2C, *r* = 0.338, *P* = 0.115) or non-stressful events (*r* = −0.056, *P* = 0.666), and age was not significantly related to either stressful events (Fig. 2A, *r* = 0.145, *P* = 0.509) or non-stressful events (Fig. 2B, *r* = −0.045, *P* = 0.837). Therefore, a multivariate linear regression analysis was used to evaluate only stress/cortisol interactions in depression, as they were both related to depressive behaviour. The other two factors (age and non-stressful events) were excluded from this interactive analysis, because neither of them was significantly related to depressive behaviour. The results showed that the main effects of both stressful events (Table 1, *b = *−0.069, SE = 0.052, *t* = −1.318, *P* = 0.203) and cortisol (Table 1, *b = *0.194, SE = 0.350, *t* = −0.533, *P* = 0.583) were not significant, but the interaction between them was found to have a significant effect on depressive behaviour (Table 1, *b = *0.018, SE = 0.006, *t* = 3.049, *P* = 0.006).

### Differences between control monkeys and depressed monkeys

**Figure 4:**
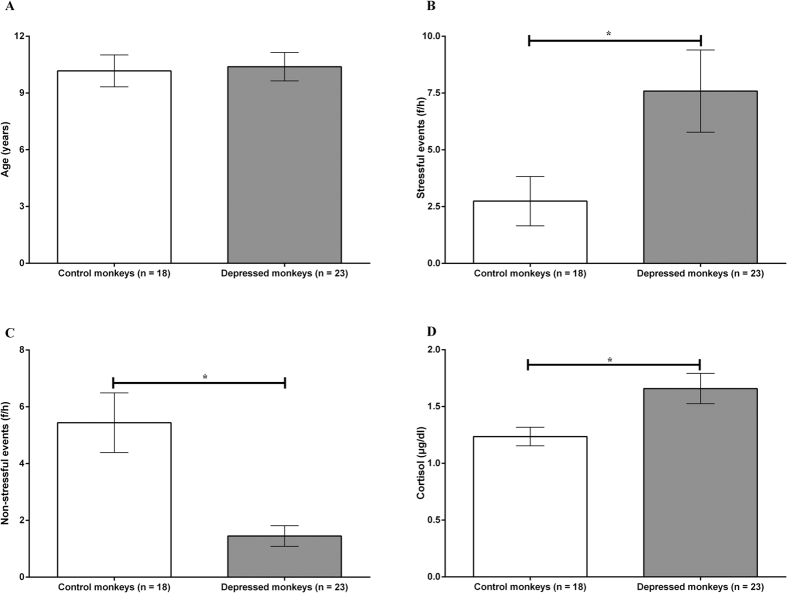
Differences between control monkeys and depressed monkeys. (**A**) On the x-axis, individuals are divided into control monkeys (n = 18) and depressed monkeys (n = 23). The y-axis represents the monkeys’ ages (years). (**B**) On the x-axis, individuals are divided into control monkeys (n = 18) and depressed monkeys (n = 23). The y-axis represents mean frequencies of stressful events that the monkeys experienced per hour (f/h). (**C**) On the x-axis, individuals are divided into control monkeys (n = 18) and depressed monkeys (n = 23). The y-axis represents the mean frequencies of non-stressful events that the monkeys experienced per hour (f/h). (**D**) On the x-axis, individuals are divided into control monkeys (n = 18) and depressed monkeys (n = 23). The y-axis represents the mean levels of hair cortisol (μg/dl).

There were 18 monkeys who never displayed depressive behaviour and were regarded as controls for comparison with depressed animals (n = 23). The results of the one-way ANOVA showed that there was no significant difference in age between the depressed monkeys and controls (Fig. 4A, *F* = 0.039, *P* = 0.844). Compared to the control monkeys, depressed monkeys experienced higher frequencies of stressful events (Fig. 4B, *F* = 7.943, *P* = 0.008) and lower frequencies of non-stressful events (Fig. 4C, *F* = 15.448, *P* = 0.0003). Furthermore, the depressed monkeys secreted more cortisol in hair than controls (Fig. 4D, *F* = 6.325, *P* = = 0.016).

## Discussion

In humans, stress is associated with the onset of depressive episodes and the severity and relapse of depression^7^. Hypercortisolism is often used as a marker of stress, as it reflects the function of the HPA axis and is a well-documented symptom in depression^15^. Evidence for the role of hypercortisolism in depression has been identified as elevated mean 24-h serum cortisol concentrations and increased 24-h urinary cortisol excretion^18^,^31^. However, the interrelationships among stress, cortisol and depression are not completely understood. Hypercortisolism is only present in 25–30% of depressed patients^32^, and some studies found no relationship between elevated cortisol secretion and major depression^33^. Other studies have even reported lower cortisol concentrations in depressed patients^34^.

A variety of laboratory-based studies have reported that social stress results in elevated cortisol levels^14^. However, there is little data on the neuroendocrine correlates of exposure to naturally occurring stressors and their association with depressive reactions. Higher cortisol levels, stressful life events, and vulnerability status have all been related to the onset of depression^35^, which has also been reported in adolescent depression^23^. However, some studies found that increased cortisol secretion was sensitive to social stress but did not mediate vulnerability to depression^20^, further complicating the associations between stress and cortisol with depression.

Several factors may account for these inconsistencies in the literature. First, the in/outpatient status of the depressed patients included in these studies varied, and research is constrained by a lack of studies that have evaluated outpatient populations, as these populations comprise the majority of patients with major depression. Second, the patients in several of these studies were on antidepressant medication, which is known to influence cortisol secretion during^36^ and after antidepressant withdrawal^37^. Third, cortisol secretion in depressed patients is different depending on the specified subgroups of depression. Cortisol hypersecretion is more pronounced in bipolar- and melancholic-depressed patients^38^, while patients with longstanding and recurrent depressive episodes often exhibit decreased serum cortisol levels^39^ or decreased urinary cortisol levels^40^. In fact, hypercortisolaemia and hypocortisolaemia are both related to depression, depending on the specific depression subtype^41^. Fourth, cortisol measured in blood or urine is subject to circadian effects. Cortisol levels measured in these samples only reflect short-term responses to stress that occur over hours or days and do not assess responses to chronic stress that occur over weeks to months^29^. These four discrepancies continue to impede the understanding of the relationship between cortisol as a stress marker and depression.

The present study investigated antagonistic interactions among rhesus monkeys as indicators of stress, cortisol levels measured from hair samples of rhesus monkeys as a measure of chronic cortisol secretion, and individual huddling behaviours of the monkeys as an indicator of depression. Behavioural measurements were conducted in a controlled environment to remove confounding factors. Hair cortisol levels are considered a biomarker of chronic stress and have been used in previous studies of rhesus macaques in which hair samples were obtained to describe the relationship between cortisol and long-term stress^15^,^30^,^42^,^43^. It was shown in this study that elevated cortisol levels were positively correlated with depressive behaviours (time spent in huddled postures) exhibited by the rhesus macaques and with the number of times an animal experienced stressful events. The stress that the monkeys experienced was also related to depressive behaviours. Behaviourally, higher frequencies of stressful events (including the receipt of aggression and displays of submission) predicted longer times spent in a huddled posture. However, the other analysed events (including displays of aggression and receipt of submission) were not found to be related to huddled postures, likely because they represented stress-attenuating coping responses rather than a source of stress^44^. These findings suggest that both stress and cortisol are correlated with the depressive behaviours observed in female rhesus monkeys. Further analysis showed that stress interacted with high cortisol levels to predict the monkeys’ huddling behaviours.

In the first reported primate model of adult depression, it was observed that depressive behaviours appeared more commonly in socially stressed subordinate females than in dominant female monkeys^14^. Although stress was a predictor for depressive patterns in cynomolgus monkeys, the relationship between stress and depression was not well quantified, nor did this study assess the interrelationship and interaction between stress and cortisol in the development of depression. To our knowledge, this is the first study to quantify the relationship between stress and depressive behaviours in rhesus macaques under a controlled setting. It is also the first study to integrate biological paradigms with data on naturally occurring stressors and risks for depressive behaviours in rhesus monkeys. The use of a controlled breeding environment, standardized stressful and non-stressful events, and a measure of chronic cortisol secretion to correlate with the depressive behaviours exhibited by female macaques allowed this study to reduce the variability that often occurs in human studies.

Although no direct mechanism has been described to link stress, cortisol and depression, these findings are consistent with the notion that the adrenal gland secretes cortisol (the major glucocorticoids in humans and nonhuman primates) in response to a variety of stressors, which leads to deleterious effects on the brain that leaves the brain susceptible to depression. Since cortisol was first discovered to be neurotoxic to the hippocampus more than 40 years ago^45^, it has been widely accepted that cortisol hypersecretion may also disrupt adaptive processes by which lower cortisol levels normally interact with cortical and limbic structures to promote cognitive and emotional processing. In addition to hippocampal neuron loss, these complex interactions within the brain and with other neurotransmitter systems may result in dysfunctions of this diverse biological system due to hypercortisolism, which could have an integrated impact on the interactions among stress, cortisol and depression onset^16^.

This study did not find any correlations among age and life events, cortisol levels or the depressive behaviours exhibited by the monkeys. Age has been identified as positively associated with cortisol secretion during depression^46^, especially in women^47^ and severely depressed subjects^48^. However, it was also reported that there are no differences in basal cortisol levels between young and old depressed populations^49^, which is similar to our findings. This suggests that age may not be a major factor in the development of depression, but rather a reflection of the differences in study designs.

By measuring conflict interactions among rhesus monkeys as indicators of stress, cortisol levels in hair samples as a measure of chronic cortisol secretion, and individual huddling behaviours as an indicator of depression, in a controlled environment, this study avoided many possible confounding effects that hinder studies in humans. This is also the first study that has integrated biological paradigms with data on naturally occurring stressors involved in the development of depression. Although the results presented here represent a small sample size, the data in adult female macaques showed that cortisol hypersecretion interacted with stress to accelerate the development of depressive behaviours, which extends the current understanding of stress/cortisol interactions in depression, especially pertaining to women. As such, replication of these findings in a larger population will allow further explorations of the possible physiological mechanisms underlying stress, cortisol and depression in females.

## Additional Information

**How to cite this article**: Qin, D.-d. *et al*. Prolonged secretion of cortisol as a possible mechanism underlying stress and depressive behaviour. *Sci. Rep.* **6**, 30187; doi: 10.1038/srep30187 (2016).

## Figures and Tables

**Table 1:** Interactive effects between stress and cortisol on depressive behaviour.

Predictors	*b*	*SE*	*t*	*P*
Stress	−0.069	0.052	−1.318	0.203
Cortisol	−0.194	0.350	−0.553	0.586
Stress × cortisol	0.018	0.006	3.049	0.006

The data presented show the multivariate linear regression analysis results with main effects and interactions, where *b* represents the slope of the linear equation.
